# 

**Published:** 1882-02-20

**Authors:** 


### TABLE OF CONTENTS.

ORIGINAL AND SELECTED ARTICLES.

Viburnum Prunifolium, by Thomas F. Houston, M. D., of Clarkesville, Ga., 41. Rhus Toxicodendron—(Poison Oak), by R. L. Hinton, M. D., of Arkansas; Abstract of a Clinical Lecture on the Recognition and Treatment of the Early Stages of Pott's Disease. by D. Hayes Agnew, M. D., of Penn, 43. Vaccinal Syphilis, 46. Practical Observations in Typhoid Fever, by H. V. Ferrell, M. D., of St. Louis, Mo., 47. Coccobacteria in Purulent Otorrhœa, by C. Lambert, M. D., of Indiana, 50. Sponge Grafting, 52. How to Amputate a Leg, by Theodore A. McGraw, M. D., of Michigan, 54. A Sim le Way of Performing Optico-Ciliary Neurotomy, by Jos. A. White, M. D., of Virginia, 56. Vaccination and Vaccinization, 57. Prize Essay, 58.

ABSTRACTS AND GLEANINGS.

Susceptibility to Variola; Small-Pox Pustulation in the Fœtus in Utero, 59. The Bone-Conduction of Sound, 60. Cold App ications in Typhoid Fever, 61. Cough and Phthisis, 62. Night Sweats, 63. Does Vaccination Protect, 64. J. J. Connor, M. D.; The Immediate Arrest of Bleeding from the Nose, 65. Expressing the Placenta, 66. Cutaneous Eruptions Caused by the use of certain medicines; Hypodermic Injection of Water in the Treatment of Pain, 67. The Specific Germ of Gonorrhœal Pus, 68. Manipulation of the Scapula in Dislocation of the Shoulder, 69 Calomel and Chlorate of Potash; Rattlesnake Poison; Dr. R. Fowler, of Ednaville, Texas, 70.

SCIENTIFIC ITEMS.

The Mosquito as a Carrier of Disease; Telephone Sound of Voices, 71. Ancient Division of Time; The Dentaphone; The Pennsylvania Railroad, 72.

PRACTICAL NOTES AND FORMULÆ.

Preston's Salts; Anise-seed Soothing Cordia'; Cathartic Enema, 73. Formulæ Used in the New York Hospital—for external use, 74. Dewee's Carminative; Zimmerman's Decoction; For Diphtheria, 75.

EDITORIALS AND MISCELLANEOUS.

Wm. R. Warner & Co.; Non-Humanized Vaccine Virus; Parke, Davis & Co.; Reed & Carnrick; Nestle's Milk Food; Fellows' Hypophosphites; Anglo-Swiss Milk Food; Small-Pox, 76. Transactions Medical Association of Georgia, 77. Arkansas State Medical Society; Book Notices, 78. Receipts; Special Notices, 80.

